# Immune-inflammation index as prognostic markers in metastatic castration-resistant prostate cancer: a systematic review and meta-analysis

**DOI:** 10.3389/fonc.2026.1806929

**Published:** 2026-04-27

**Authors:** Zitong Qin, Runzhang Liu, Suoshi Jing, Chenhao Guo, Weiping Li

**Affiliations:** 1 The First Clinical Medical College of Lanzhou University, Lanzhou, China; 2 Department of Urology, The First Hospital of Lanzhou University, Lanzhou, China

**Keywords:** lymphocyte-to-monocyte ratio, meta-analysis, metastatic castration-resistant prostate cancer, neutrophil-to-lymphocyte ratio, platelet-to-lymphocyte ratio, systemic immune-inflammation index

## Abstract

**Introduction:**

Systemic inflammation is closely linked to tumor progression and immune dysfunction. Simple blood-derived immune-inflammatory indices may provide cost-effective prognostic stratification for metastatic castration-resistant prostate cancer.

**Methods:**

This systematic review and meta-analysis followed PRISMA 2020 and was registered in PROSPERO. Electronic databases, including Embase, PubMed, Web of Science, and the Cochrane Library, were queried from inception until May 2025. Eligibility was restricted to research comparing high and low strata of the systemic immune-inflammation index (SII), lymphocyte-to-monocyte ratio (LMR), and ratios of neutrophils or platelets to lymphocytes, provided they reported survival outcomes (OS and/or PFS) via hazard ratios (HRs) and 95% confidence intervals (CIs). Random-effects models were applied according to heterogeneity; subgroup, sensitivity, and publication-bias analyses were performed.

**Results:**

36 studies involving 10,325 patients with mCRPC were included. Elevated NLR was associated with worse OS (pooled HR = 1.77, 95% CI 1.59–1.97) and PFS (pooled HR = 1.51, 95% CI 1.31–1.75). Higher PLR predicted shorter OS (pooled HR = 1.57, 95% CI 1.35–1.84) and PFS (pooled HR = 1.45, 95% CI 1.13–1.87). Higher LMR was associated with improved OS (pooled HR = 0.45, 95% CI 0.31–0.67), while higher SII predicted worse OS (pooled HR = 2.22, 95% CI 1.86–2.66). Subgroup analyses (where feasible) supported the robustness of NLR/PLR associations across several clinical strata, though some subgroups were limited by small numbers.

**Conclusion:**

Blood-based immune-inflammatory indices (NLR, PLR, SII, LMR) are significantly associated with survival outcomes in mCRPC. These readily available markers may aid risk stratification and inform individualized management, while prospective validation and standardized cut-offs remain needed.

**Systematic Review Registration:**

https://www.crd.york.ac.uk/prospero/display_record.php?RecordID=1291003, identifier CRD420261291003.

## 1 Introduction

Prostate cancer poses a major global health challenge for men, ranking among the leading malignancies in terms of both incidence and mortality (1). Prostate cancer’s global burden involved approximately 1.4 million newly diagnosed cases in 2022, a figure representing 7.3% of all malignancies; this disease further contributes to about 375,000 deaths on an annual basis (2). The clinical course of prostate cancer typically progresses from localized tumors to the metastatic castration-resistant stage, characterized by persistent disease progression despite serum testosterone being at castrate levels (3, 4). Despite improvements in early detection and primary therapeutic strategies for localized disease, a significant subset of patients will ultimately see their condition progress to a castration-resistant state, signifying a transition into a clinical phase with restricted therapeutic avenues and a markedly poorer outlook. The prognosis for metastatic castration-resistant prostate cancer (mCRPC) patients is severe, with a traditional median overall survival ranging from approximately 9 to 30 months (5). Despite the emergence of various treatments in recent years, including novel endocrine therapies, chemotherapy, and targeted radiotherapies, mCRPC remains incurable, and patient outcomes show substantial heterogeneity (6). Therefore, there is an urgent need for prognostic biomarkers to guide individualized treatment.

Inflammation participates in the entire process of tumorigenesis and development, including initiation, promotion, progression, and metastasis (7, 8). Chronic inflammation can drive tumor progression through multiple mechanisms, while tumor cells can actively create and maintain a pro-inflammatory microenvironment, with the two reinforcing each other (9). In summary, chronic inflammation is a key factor throughout tumor occurrence, development, treatment response, and prognostic assessment. In prostate cancer, it serves as an important biological basis driving tumor progression and drug resistance. Derived serological indicators have become important clinical prognostic tools. In cancer immunotherapy, peripheral blood inflammatory markers hold significant predictive value. Among these, the neutrophil-to-lymphocyte ratio (NLR) is the most extensively studied indicator, reflecting the balance between systemic inflammation and anti-tumor immunity (10, 11). A low NLR typically suggests better immunotherapy responses (12). The platelet-to-lymphocyte ratio (PLR) incorporates platelet count, linking inflammation and thrombosis, and participates in tumor metastasis and immune escape (13, 14). The systemic immune-inflammation index (SII) is an integrated parameter calculated by multiplying the neutrophil and platelet counts and subsequently dividing the result by the lymphocyte count. This composite marker is thought to offer a more holistic representation of a patient’s immunological and inflammatory profile (15, 16). The lymphocyte-to-monocyte ratio (LMR) reflects the balance between adaptive immunity and the monocyte/macrophage system, with a low LMR often predicting poor prognosis (17). This balance is mechanistically supported by the complex interplay between lymphocytes and macrophages in the tumor microenvironment. These indicators are all easily accessible potential prognostic tools.

In the field of prostate cancer research, existing studies on inflammatory markers have limitations such as heterogeneous patient populations and inconsistent marker definitions. This study aims to conduct a meta-analysis to evaluate the prognostic significance of NLR, PLR, SII, and LMR in mCRPC patients, in order to provide robust support for stratifying patient risk and tailoring therapeutic strategies.

## 2 Methods

### 2.1 Literature search

This systematic review was conducted and reported in strict accordance with the Preferred Reporting Items for Systematic Reviews and Meta Analyses (PRISMA 2020) statement (PRISMA checklist provided as **Supplementary Table 3**), and the research protocol was registered on the International Prospective Systematic Evaluation Registry (PROSPERO: CRD420261291003) (18). The search protocol was designed and refined by two researchers (QZT and LRZ), who separately established a combination of MeSH terms and free-text words. These parameters were utilized to interrogate multiple electronic repositories, specifically PubMed, the Cochrane Library, Embase, and Web of Science. We examined all indexed literature from the point of database inception through May 28, 2025. This search was conducted by incorporating an exhaustive array of descriptors, such as (((“Lymphocytes”[Mesh]) OR (((Lymphocyte) OR (Lymphoid Cells)) OR (Lymphoid Cell))) AND (Ratio OR index OR score)) AND ((“Prostatic Neoplasms, Castration-Resistant”[Mesh]) OR (((((((((((((((((((((((((((((Castration-Resistant Prostatic Neoplasm) OR (Androgen-Independent Prostatic Neoplasms)) OR (Androgen Independent Prostatic Neoplasms)) OR (Androgen-Insensitive Prostatic Neoplasms)) OR (Androgen Insensitive Prostatic Neoplasms)) OR (Androgen-Resistant Prostatic Neoplasms)) OR (Androgen Resistant Prostatic Neoplasms)) OR (Castration-Resistant Prostatic Neoplasms)) OR (Castration Resistant Prostatic Neoplasms)) OR (Hormone Refractory Prostatic Neoplasms)) OR (Androgen-Independent Prostatic Neoplasm)) OR (Androgen-Insensitive Prostatic Neoplasm)) OR (Androgen-Resistant Prostatic Neoplasm)) OR (Castration-Resistant Prostatic Cancers)) OR (Androgen-Insensitive Prostatic Cancer)) OR (Androgen Insensitive Prostatic Cancer)) OR (Androgen-Resistant Prostatic Cancer)) OR (Androgen Resistant Prostatic Cancer)) OR (Castration-Resistant Prostatic Cancer)) OR (Castration Resistant Prostatic Cancer)) OR (Hormone Refractory Prostatic Cancer)) OR (Androgen-Independent Prostatic Cancer)) OR (Androgen Independent Prostatic Cancer)) OR (Androgen-Insensitive Prostatic Cancers)) OR (Androgen-Resistant Prostatic Cancers)) OR (Androgen-Independent Prostatic Cancers)) OR (CRPC)) OR (Castration Resistant Prostate Cancer)) OR (Castration-Resistant Prostate Cancer))). The specific methodology and terms used for the literature survey are delineated in **Supplementary Table 1**.

### 2.2 Study selection

Studies eligible for inclusion in our analysis should meet the following criteria: (1) Patients were diagnosed with mCRPC through pathologic observation;(2) Studies focused on the association between blood-derived inflammatory indices and clinical survival outcomes (OS and PFS) in the context of mCRPC; (3) articles reported hazard ratios (HRs) and 95% confidence intervals (CIs), either explicitly or as values derivable from the provided statistics; (4) participants were categorized into high- versus low-level groups according to specific cut-off thresholds; and (5) the study was published as a full-text article. Conversely, studies were excluded if they were: (1) non-original reports such as reviews, editorials, conference abstracts, case reports, or letters; (2) lacking sufficient data to estimate HRs and 95% CIs; (3) devoid of survival outcome data; or (4) based on duplicate or overlapping patient cohorts.

The literature screening was conducted independently by two investigators (QZT and LRZ), who first assessed titles and abstracts before performing a comprehensive full-text review to confirm eligibility. Any discrepancies arising during this selection phase were settled through mutual discussion to reach a consensus.

### 2.3 Data extraction

To ensure accuracy, the data extraction process involved two independent researchers QZT and LRZ. Any inconsistencies identified were subsequently addressed and finalized by consensus of the entire author team. Extracted information included the name of the first author, study period, region, study design, Treatment, Detection timing, member of patients, Mean/median Age, Mean/median BMI (kg/m²), Mean/median PSA(ng/mL), TNM stage, Marker, Cut-off, OS and PFS. To ensure data uniformity, for studies presenting the monocyte-to-lymphocyte ratio (MLR), we mathematically transformed the results into LMR. This was achieved by inverting the hazard ratios and swapping the lower and upper boundaries of the 95% confidence intervals (19).

### 2.4 Quality assessment

Study quality was appraised with the Newcastle-Ottawa Quality Assessment Scale (NOS) (20). As a tool for observational studies, the NOS allocates points (up to 9) based on selection, comparability, and outcome assessment. Consistent with prior literature, a score ranging from 6 to 9 was defined as indicative of high study quality (21).

### 2.5 Statistical analysis

To evaluate the prognostic impact of NLR, PLR, SII, and LMR on PFS or OS, we calculated pooled HRs with 95% CIs. Cochran’s Q test and Higgins I^2^ statistic were utilized for measuring heterogeneity (22). Heterogeneity was deemed significant if P<0.1 or I^2^>50%. To calculate pooled estimates across the included studies, the meta-analysis was performed using a random-effects methodology. Additionally, we evaluated the stability of our results and explored potential drivers of inter-study variability by performing stratified analyses and sensitivity tests. To detect evidence of publication bias, both Egger’s test and a qualitative inspection of funnel plot symmetry were conducted. A threshold of P < 0.05 was utilized to define significant findings across all analytical procedures. The statistical computations were performed using STATA 15.0 and Review Manager 5.4.

## 3 Results

### 3.1 Study characteristics

**Figure 1:**
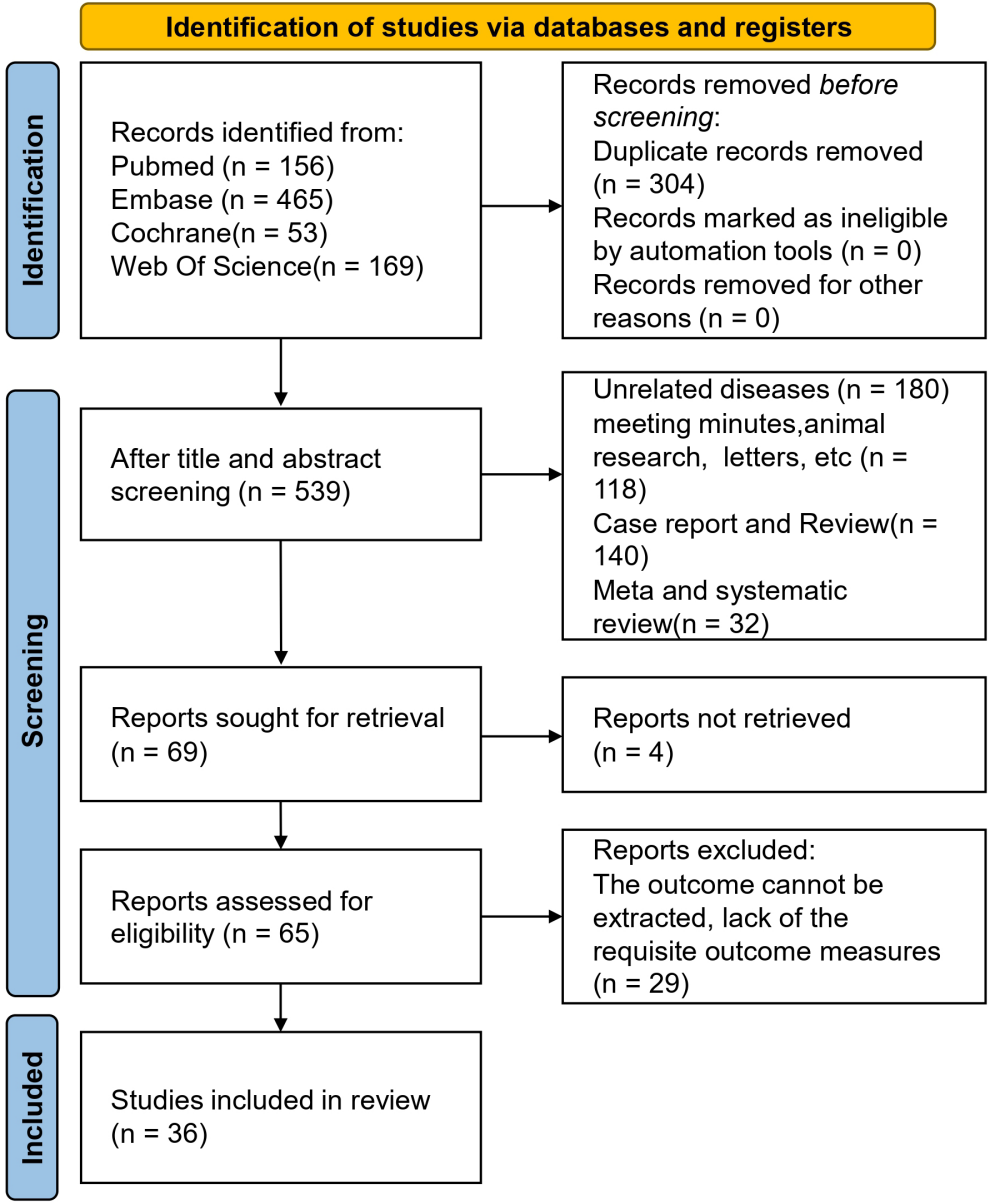
Flow chart of literature screening.

The initial database query yielded 843 records. Following the elimination of 304 duplicates, we screened the titles and abstracts of the remaining entries, which led to the removal of 470 articles. Subsequently, 69 full-text articles were evaluated for eligibility. Of these, 29 were rejected, largely because of unextractable results or the absence of necessary prognostic endpoints. In the end, 36 studies involving a cumulative cohort of 10,325 patients were enrolled in the meta-analysis (19, 23–57) (**Figure 1**). The studies spanned a wide time period from 1998 to 2025. Of the included studies, 28 were retrospective and 8 were prospective cohorts, with all studies published in English.

Geographically, the studies originated from a wide range of regions, including Italy, Germany, South Korea, the United States, Spain, China, Japan, Turkey, Canada, the United Kingdom, and international multicenter collaborations. Among these, Italy (7 studies), China (5 studies), Germany (4 studies), and the United States (4 studies) had the highest number of studies.

Regarding treatment modalities, the studies primarily focused on various systemic therapies for mCRPC, including docetaxel chemotherapy, novel endocrine therapies (abiraterone, enzalutamide), radium-223, cabazitaxel, and radioligand therapy. Most studies measured inflammatory markers at baseline treatment (labeled as “Baseline”), while a few conducted measurements during treatment (such as at 4 weeks, 12 weeks) or at the time of CRPC diagnosis/progression. The sample sizes varied widely, ranging from a minimum of 41 patients (Jiang et al., 2021) to a maximum of 2,230 patients (van Soest et al., 2014). The median age of patients was concentrated between 66 and 75 years. The table focused on the prognostic value of several systemic inflammatory markers, including NLR, PLR, SII, and LMR. The vast majority of studies reported NLR and its cutoff values, while fewer studies reported cutoff values for PLR, SII, and LMR, with 8, 6, and 4 studies, respectively. The primary outcome measures were OS and/or PFS. **Table 1** presents result.

**Table 1:** Basic characteristics of the included literature.

Author	Study period	Sources of patients	Study type	Treatment	Detection timing	Sample size	Age	Mean/median PSA(ng/L)	NLR cut-off	PLR cut-off	SII cut-off	LMR cut-off	Outcome	Quality score
Bauckneh et al.2022 (19)	2013-2020	Italy	Cohort (Retrospective)	Radiotherapy	Baseline	519	74	54	3.1	145.9	768.8	2.8	OS	7
Boegemann et al.2017 (23)	2009-2015	Germany	Cohort (Retrospective)	ADT	Baseline	113	70	134	5	NA	NA	NA	OS&PFS	7
Boegemann et al.2019 (24)	2009-2015	Germany	Cohort (Retrospective)	ADT	Baseline	117	70	122	3.7	NA	NA	NA	OS	7
Buttigliero et al.2017 (25)	2004-2016	Italy	Cohort (Retrospective)	Chemotherapy	Baseline	110	68	47	3	NA	NA	NA	OS&PFS	9
Choi et al.2018 (26)	2014-2017	Korea	Cohort (Retrospective)	ADT	Baseline	113	73	43.6	3	NA	NA	NA	OS&PFS	7
Chong et al.2021 (27)	2018-2021	USA	Cohort (Prospective)	Combined Therapy	Baseline	63	70.9	8.8	2.65	155.54	NA	NA	OS&PFS	6
Conteduca et al.2016 (28)	2012-2014	Italy/UK	Cohort (Retrospective)	ADT	4 weeks	193	73.1	NA	3	NA	NA	NA	OS&PFS	6
Conteduca et al.2018 (29)	2011-2016	Italy	Cohort (Retrospective)	ADT	Baseline	551	75	46.8	3	NA	NA	NA	OS	8
Conteduca et al.2019 (30)	2011-2016	Italy	Cohort (Retrospective)	ADT	Baseline	105	74	29.3	3	NA	NA	NA	OS	6
Donate-Moreno et al.2020 (31)	Until 2018	Spain	Cohort (Prospective)	ADT	Baseline	80	72.7	NA	3	150	53500	3	OS	8
España et al.2020 (32)	2011-2014	Spain	Cohort (Retrospective)	ADT	Baseline	104	74	284.6	4	NA	NA	NA	OS	6
Fan et al.2017 (33)	2013-2017	China	Cohort (Retrospective)	Combined Therapy	Baseline	104	72	16.7	3	NA	200	NA	OS&PFS	6
Fujiwara et al.2020 (34)	2012-2018	Japan	Cohort (Retrospective)	ADT	Baseline	184	75	8.89	3	NA	NA	NA	OS	8
Jiang et al.2021 (35)	2016-2019	China	Cohort (Retrospective)	Combined Therapy	Baseline	41	73.4	251.1	3	NA	NA	NA	OS&PFS	7
Koo et al.2019 (36)	2009-2017	Korea	Cohort (Retrospective)	Combined Therapy	At CRPC diagnosis	303	66.5	69.2	2.5	NA	NA	NA	PFS	8
Linton et al.2013 (37)	2007-2009	USA/Russia	Cohort (Prospective)	Combined Therapy	Baseline	184	NA	NA	5	NA	NA	NA	OS	8
Lolli et al.2016 (38)	2011-2015	Italy	Cohort (Retrospective)	ADT	Baseline	230	74	NA	3	210	535	NA	OS	7
Lorente et al.2015 (39)	TROPIC trial period	International	Cohort (Prospective)	Combined Therapy	Baseline	755	67	NA	3	NA	NA	NA	OS&PFS	7
Man et al.2019 (40)	2010-2018	China	Cohort (Retrospective)	Chemotherapy	Baseline	179	70	62.89	3	210	535	NA	OS	8
Meisel et al.2022 (41)	PROSELICA trial period	International	Cohort (Prospective)	Chemotherapy	Baseline	1200	NA	35.9	3&3.8	NA	NA	NA	OS&PFS	8
Neuberger et al.2022 (42)	2010-2019	Germany	Cohort (Retrospective)	Chemotherapy	Baseline	118	72	82	3	NA	NA	NA	OS	6
Nuhn et al.2014 (43)	1998-2010	USA	Cohort (Retrospective)	Chemotherapy	Baseline	238	68.3	NA	3	NA	NA	NA	OS	8
Onal et al.2018 (44)	2012-2017	Turkey	Cohort (Retrospective)	ADT	Baseline, 4w, 12w	102	71	47	3.1	163	NA	NA	OS&PFS	7
Pei et al.2017 (45)	2009-2016	China	Cohort (Retrospective)	Chemotherapy	Baseline	111	71	150	3.3	NA	NA	NA	OS&PFS	8
Pisano et al.2021 (46)	2010-2018	Italy	Cohort (Retrospective)	Combined Therapy	Baseline	225	73	35.7	3	128&190	NA	NA	OS&PFS	6
Sahin et al.2023 (47)	2015-2023	Turkey	Cohort (Retrospective)	Radiotherapy	Baseline	61	69.8	36.6	2.7	134.27	570.39	2.44	OS	8
Sonpavde et al.2014 (49)	2008-2010	International	Cohort (Prospective)	Combined Therapy	Baseline	873	68	130	2.5&5	NA	NA	NA	OS	9
Steffens et al.2025 (48)	2010-2023	Germany	Cohort (Retrospective)	Chemotherapy	Before chemotherapy	153	72	63.3	3	NA	NA	NA	OS	8
Templeton et al.2014 (50)	2001-2012	Canada/UK	Cohort (Retrospective)	Chemotherapy	Baseline	357	71	162	3	NA	NA	NA	OS	8
Uemura et al.2017 (51)	2014-2016	Japan	Cohort (Retrospective)	Chemotherapy	Before chemotherapy	47	71.4	150.3	3.83	NA	NA	NA	OS	6
Uzun et al.2025 (52)	2015-2023	Turkey	Cohort (Retrospective)	Combined Therapy	At CRPC progression	113	73	NA	NA	NA	700	NA	OS	7
van Soest et al.2014 (53)	VENICE (NCT00519285) period	International	Cohort (Prospective)	Combined Therapy	Baseline	2230	68	86.8	2	NA	NA	NA	OS	7
Wit et al.2021 (54)	CARD study (NCT02485691) period	International	Cohort (Prospective)	Chemotherapy	Baseline	125	69.6	450	3.38	NA	NA	NA	OS	8
Wu et al.2018 (55)	2009-2016	China	Cohort (Retrospective)	Combined Therapy	Before chemotherapy	71	71	199.2	3.3	NA	NA	NA	OS	7
Yamada et al.2020 (56)	2007-2018	Japan	Cohort (Retrospective)	Combined Therapy	At CRPC progression	196	75	397.15	2.04	66.88	NA	NA	OS	7
Yamamoto et al.2023 (57)	2015-2021	Japan	Cohort (Retrospective)	Chemotherapy	Baseline	57	70	42.5	3.8	NA	NA	NA	OS	6

### 3.2 Study quality

The quality appraisal revealed that all 36 included articles maintained high methodological standards, achieving NOS scores ranging from 6 to 9 (**Supplementary Table 2**).

### 3.3 Meta-analysis results

#### 3.3.1 Prognostic value of NLR for OS and PFS in mCRPC

##### 3.3.1.1 NLR and OS

**Figure 2:**
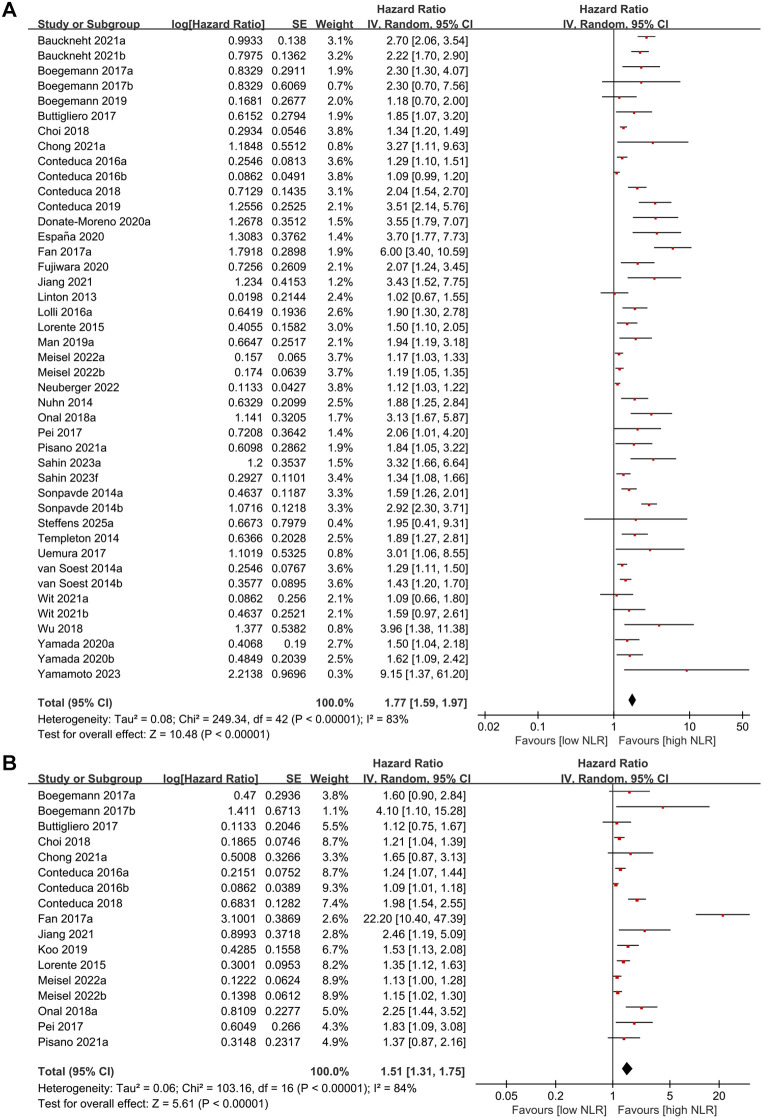
**(A)** Forest plots for the association between NLR and OS. **(B)** Forest plots for the association between NLR and PFS.

We initially evaluated the association linking NLR levels to overall survival. The meta-analysis pooled data from 43 independent studies. Most analyses provided the HR for OS using baseline NLR values, while a minority reported NLR data post-treatment (such as at 4, 8, or 12 weeks). The pooled results demonstrated that a higher NLR was a significant adverse prognostic factor for OS, associated with shorter OS in patients with mCRPC (pooled HR = 1.77, 95% CI: 1.59–1.97; p<0.00001, **Figure 2A**). Subgroup analyses demonstrated that the adverse prognostic impact of elevated NLR on OS was consistently observed across different study designs (prospective and retrospective), age groups (>70 vs. ≤70 years), geographic regions (Asia, Europe, America, and multicenter studies), and treatment modalities (ADT, chemotherapy, radiotherapy, and combination therapy). The association remained statistically significant regardless of baseline PSA levels or the NLR cut-off values applied (**Table 2**). Although substantial heterogeneity persisted in several subgroups, the direction of effect was uniform, supporting the robustness of NLR as a negative prognostic marker for OS in mCRPC.

**Table 2:** Subgroup analysis of pooled HRs for OS and PFS according to NLR and PLR in mCRPC.

*Subgroups*	NLR-OS (Baseline)				NLR-PFS (Baseline)				PLR-OS (Baseline)			
	Study	HR [95%CI]	P value	*I^2^*	Study	HR [95%CI]	P value	*I^2^*	Study	HR [95%CI]	P value	*I^2^*
Total	43	1.77 [1.59-1.97]	<0.00001	83%	17	1.51 [1.31-1.75]	<0.00001	84%	10	1.57 [1.35-1.84]	<0.00001	7%
Study design												
Prospective	12	1.51 [1.27-1.79]	<0.00001	83%	4	1.19 [1.08-1.30]	0.0002	19%	2	2.18 [1.28-3.69]	0.004	0%
Retrospective	31	1.94 [1.69-2.24]	<0.00001	84%	13	1.73 [1.39-2.14]	<0.00001	88%	8	1.52 [1.28-1.81]	<0.00001	14%
Mean/median age												
>70y	24	2.03 [1.73-2.40]	<0.00001	87%	10	1.81 [1.41-2.32]	<0.00001	90%	8	1.59 [1.36-1.86]	<0.00001	0%
≤70y	16	1.69 [1.43-1.99]	<0.00001	70%	5	1.39 [1.19-1.64]	<0.0001	10%	2	1.58 [0.70-3.58]	0.27	71%
Region												
Asia	11	2.27 [1.67-3.10]	<0.00001	77%	5	2.62 [1.36-5.06]	0.004	93%	2	1.28 [0.94-1.74]	0.12	0%
Europe	19	1.91 [1.59-2.29]	<0.00001	87%	8	1.46 [1.18-1.80]	0.0005	79%	7	1.66 [1.39-1.98]	<0.00001	4%
America	2	2.02 [1.37-2.97]	0.0003	0%	1	1.65 [0.87-3.13]	0.13	/	1	2.38 [0.88-6.44]	0.09	/
Multicenter	9	1.46 [1.23-1.75]	<0.0001	85%	3	1.18 [1.08-1.29]	0.0004	24%	NA	NA	NA	NA
Treatment												
ADT	15	1.89 [1.55-2.29]	<0.00001	87%	9	1.44 [1.21-1.71]	<0.0001	77%	6	1.54 [1.22-1.93]	0.0002	0%
Chemotherapy	18	1.41 [1.26-1.58]	<0.00001	63%	6	1.24 [1.09-1.40]	0.0009	46%	1	1.07 [0.62-1.85]	0.81	/
Radiotherapy	4	2.16 [1.45-3.21]	0.0001	85%	NA	NA	NA	NA	2	1.87 [1.46-2.40]	<0.00001	0%
Combined Therapy	2	5.00 [3.16-7.92]	<0.00001	4%	2	5.70 [0.42-78.14]	0.19	98%	NA	NA	NA	NA
Mean/median PSA												
>100	16	1.87 [1.55-2.25]	<0.00001	64%	4	1.96 [1.41-2.72]	<0.0001	0%	1	1.38 [0.96-2.01]	0.09	/
≤100	20	1.85 [1.58-2.16]	<0.00001	87%	10	1.64 [1.30-2.08]	<0.0001	89%	7	1.61 [1.27-2.03]	<0.0001	24%
NLR cut-off									PLR cut-off			
>3	15	2.03 [1.52-2.71]	<0.00001	84%	5	1.71 [1.16-2.52]	0.007	72%	5	1.38 [1.06-1.82]	0.02	13%
≤3	28	1.68 [1.50-1.87]	<0.00001	81%	12	1.50 [1.26-1.78]	<0.00001	88%	5	1.70 [1.42-2.05]	<0.00001	0%

##### 3.3.1.2 NLR and PFS

Similarly, the association between NLR and PFS was analyzed across 17 studies. Echoing the findings from the OS analysis, a higher NLR was a significant adverse prognostic factor for PFS, associated with shorter PFS in patients with mCRPC (pooled HR = 1.51,95% CI:1.31–1.75; p<0.00001) (**Figure 2B**). Subgroup analyses for PFS showed that higher NLR was consistently associated with shorter PFS across study designs, age strata, regions, and major treatment regimens, including ADT and chemotherapy. While some subgroups (such as American cohorts and combination therapy) did not reach statistical significance, these analyses were limited by small sample sizes. Overall, subgroup results reinforced the prognostic relevance of elevated NLR for both OS and PFS in mCRPC patients (**Table 2**).

#### 3.3.2 Prognostic value of PLR for OS and PFS in mCRPC

##### 3.3.2.1 PLR and OS

**Figure 3:**
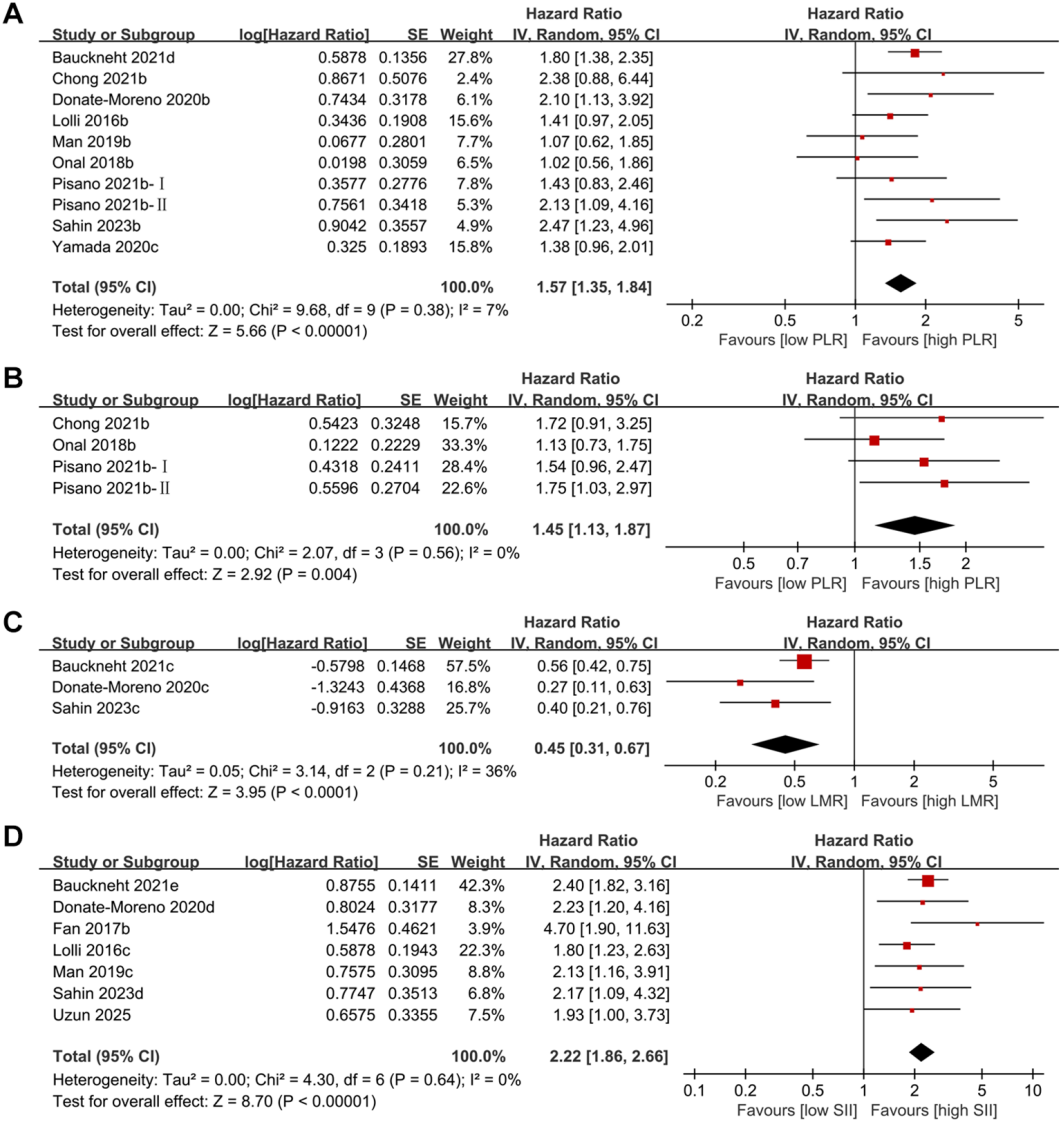
**(A)** Forest plots for the association between PLR and OS. **(B)** Forest plots for the association between PLR and PFS. **(C)** Forest plots for the association between LMR and OS. **(D)** Forest plots for the association between SII and OS.

10 studies specifically evaluated the prognostic impact of PLR on overall survival. Heterogeneity among the studies was low (I² =7%). The pooled results indicated that a higher PLR was a significant adverse prognostic factor for OS, associated with shorter OS in patients with mCRPC (pooled HR = 1.57, 95% CI: 1.35–1.84; p<0.00001) (**Figure 3A**). Subgroup analyses indicated that an elevated PLR was generally associated with poorer OS across most predefined strata. The adverse prognostic effect of PLR was consistently observed in both prospective and retrospective studies, in patients aged >70 years, and among those receiving ADT or radiotherapy. By geographic region, the association was significant in European studies but did not reach statistical significance in Asian or American cohorts, likely due to the limited number of included studies. Considerable heterogeneity was noted in some subgroups, particularly among younger patients and non-European populations (**Table 2**).

##### 3.3.2.2 PLR and PFS

Following the analysis of PLR and OS, we extended our analysis to examine the prognostic impact of PLR on PFS. This analysis included a total of 4 studies. The pooled results showed that in patients with mCRPC, a higher PLR was a significant adverse prognostic factor for PFS, associated with shorter PFS (pooled HR = 1.45, 95% CI: 1.13–1.87; p=0.004) (**Figure 3B**). No heterogeneity was observed among the studies (I²=0%). Given that merely 4 cohorts documented the impact of PLR on PFS in the mCRPC setting, conducting subgroup analyses was deemed unfeasible.

#### 3.3.3 Prognostic value of LMR for OS in mCRPC

A subset of three investigations was incorporated into our analysis to evaluate how LMR levels correlate with OS outcomes in the mCRPC population. This meta-analysis pooled data from all 3 studies, each providing the HR for LMR and OS. The pooled results showed that in patients with mCRPC, a higher LMR was a significantly favorable prognostic factor for OS, associated with longer OS (pooled HR = 0.45, 95% CI: 0.31–0.67; p < 0.0001) (**Figure 3C**). Low-to-moderate heterogeneity was observed across studies (I² = 36%). This analysis indicates that LMR is a favorable prognostic biomarker for OS in mCRPC.

#### 3.3.4 Prognostic value of SII for OS in mCRPC

We investigated the relationship between the SII and OS. This meta-analysis pooled data from 7 studies. The aggregated data revealed that an elevated SII functions as a robust negative predictor for OS, correlating with significantly reduced survival times in mCRPC patients (pooled HR = 2.22, 95% CI: 1.86–2.66; p<0.00001) (**Figure 3D**). No significant heterogeneity was observed among the studies (I²=0%). This analysis indicates that baseline SII is an adverse prognostic biomarker for OS in patients with mCRPC. Its effect is significant (HR = 2.22) and no heterogeneity was detected across all included studies (I²=0%), suggesting that SII holds important clinical reference value in predicting patient survival outcomes.

### 3.4 Sensitivity analysis

**Figure 4:**
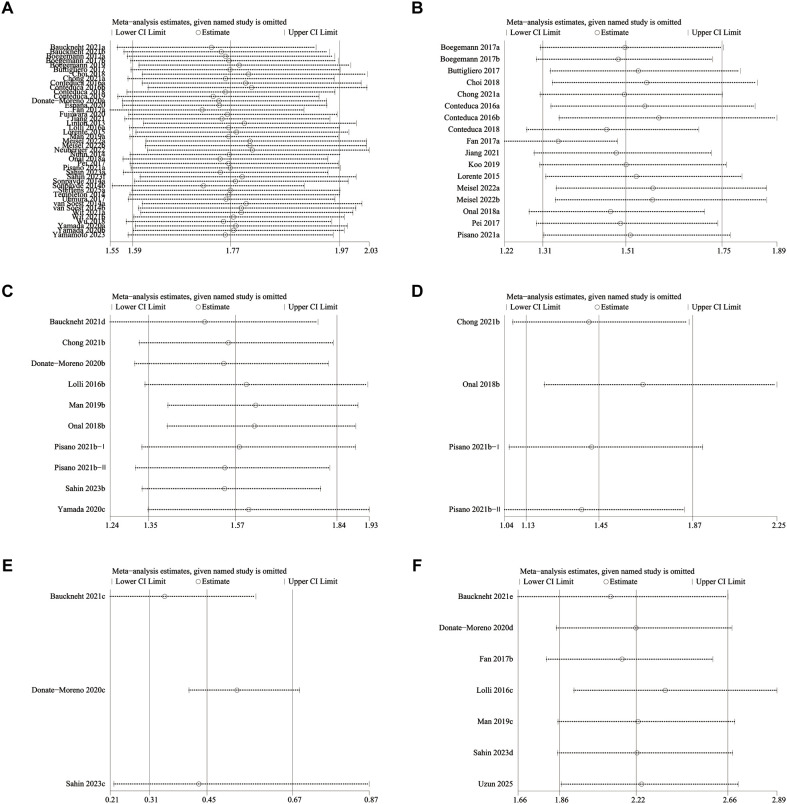
**(A)** Sensitivity analysis of NLR for OS. **(B)** Sensitivity analysis of NLR for PFS. **(C)** Sensitivity analysis of PLR for OS. **(D)** Sensitivity analysis of PLR for PFS. **(E)** Sensitivity analysis of LMR for OS. **(F)** Sensitivity analysis of SII for OS.

To validate the reliability of the prognostic associations for baseline inflammatory indices, we performed a leave-one-out sensitivity analysis. By systematically omitting one study at a time, we observed that the recalculated pooled effect sizes did not deviate significantly from the primary estimates. This confirms that the findings for NLR (OS: **Figure 4A**; PFS: **Figure 4B**), PLR (OS: **Figure 4C**; PFS: **Figure 4D**), LMR-OS (**Figure 4E**), and SII-OS (**Figure 4F**) were not driven by any individual dataset, thus supporting the overall robustness of our conclusions.

### 3.5 Publication bias

**Figure 5:**
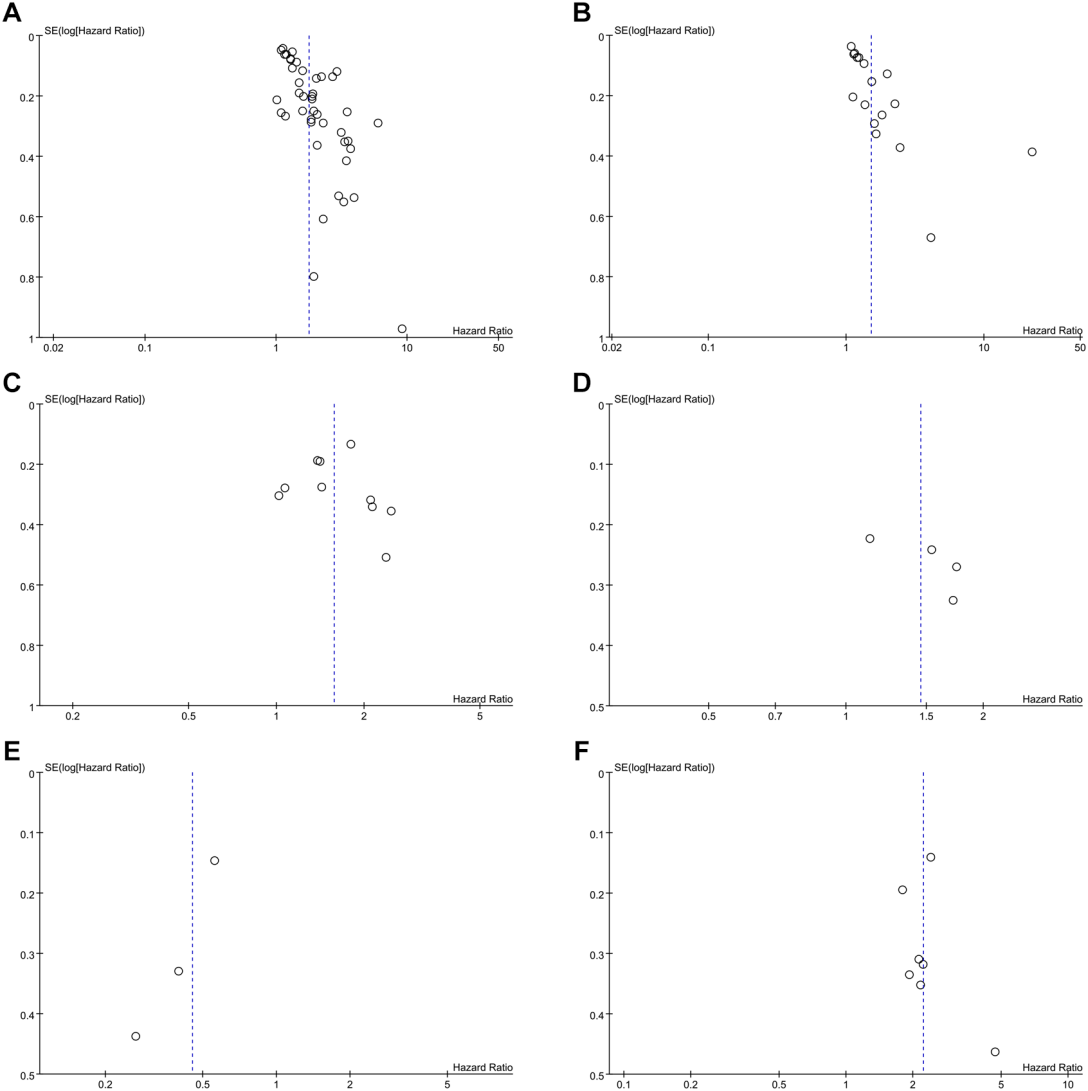
Funnel plot for the association between **(A)** NLR and OS (Egger’s test: P<0.0001), **(B)** NLR and PFS (Egger’s test: P = 0.01), **(C)** PLR and OS (Egger’s test: P = 0.782), **(D)** PLR and PFS (Egger’s test: P = 0.233), **(E)** LMR and OS (Egger’s test: P = 0.109), **(F)** SII and OS (Egger’s test: P = 0.622).

To detect potential reporting bias, we combined qualitative funnel plot symmetry assessments with quantitative analysis via Egger’s regression test. This dual approach allowed for a robust evaluation of the likelihood of selective publication within the included studies. The results indicated potential publication bias for the associations of NLR with OS (Egger: p < 0.0001) and PFS (Egger: p = 0.01), as evidenced by asymmetrical funnel plots (**Figures 5A, B**). In contrast, symmetrical funnel plots and non-significant Egger’s test results suggested no substantial publication bias for the associations of PLR with OS (Egger: p = 0.782) and PFS (Egger: p = 0.233), LMR with OS (Egger: p = 0.109), or SII with OS (Egger: p = 0.622) (**Figures 5C–F**). However, for the analysis of LMR and OS, due to the limited number of included studies (n = 3), the current statistical power may be insufficient to effectively assess the risk of publication bias. Overall, these results largely support the validity of our pooled estimates, although caution is warranted when interpreting the NLR-related findings. This asymmetry is consistent with small-study effects and selective publication, which may lead to an overestimation of the adverse prognostic effect of elevated NLR; therefore, the magnitude of the pooled HR should be interpreted cautiously.

## 4 Discussion

This systematic review and meta-analysis synthesize evidence from over 10,000 patients to definitively evaluate the prognostic utility of four systemic inflammatory indices (NLR, PLR, SII, and LMR) in the specific context of mCRPC. Our findings confirm that systemic inflammation is a critical determinant of survival in mCRPC. Specifically, elevated NLR, PLR, and SII were significantly associated with shortened OS and PFS, while a higher LMR served as a robust protective factor. Notably, the SII demonstrated the strongest predictive capability (HR = 2.22) for overall survival, suggesting that composite markers integrating multiple immune pathways potentially outperform simple dual-component ratios in predicting patient outcomes.

The NLR was the most validated marker in our study (n=43 for OS, n=17 for PFS). Our findings showed pooled HRs of 1.77 (95% CI: 1.59–1.97) for OS and 1.51 (95% CI: 1.31–1.75) for PFS, which are consistent with prior high-quality evidence and confirm that an elevated NLR independently predicts poorer survival outcomes (58, 59). The primary strength of this study lies in the demonstration of NLR’s remarkable consistency across diverse clinical scenarios. Subgroup analyses revealed that the prognostic value of NLR remains statistically significant regardless of patient age (>70 vs. ≤70), study design, or geographic region. Crucially, our analysis highlights NLR as a “pan-treatment” biomarker. We observed consistent prognostic value in patients treated with docetaxel, novel endocrine therapies like abiraterone and enzalutamide, and emerging radionuclide therapies such as Radium-223 or 177Lu-PSMA-617 (43, 53, 60, 61). This cross-modality consistency suggests that NLR captures a fundamental biological state, host immune failure, rather than a treatment-specific pharmacodynamic effect. Biologically, this ratio reflects a dual mechanism of tumor promotion: neutrophilia drives tumorigenesis via the secretion of vascular endothelial growth factor (VEGF) and matrix metalloproteinases, while neutrophil extracellular traps (NETs) can sequester circulating tumor cells to facilitate metastasis (62). Concurrently, lymphopenia indicates a depletion of cytotoxic T-cells essential for tumor surveillance (63). Thus, a high NLR serves as a proxy for a pro-tumorigenic, immunosuppressive microenvironment that blunts the efficacy of systemic therapies.

While PLR was identified as a significant prognostic factor for both OS (HR = 1.57, 95% CI: 1.35–1.84) and PFS (HR = 1.45, 95% CI: 1.13–1.87), our subgroup analysis uncovered a striking geographical disparity. The prognostic value of PLR was highly significant in European populations (HR = 1.66) but failed to reach statistical significance in Asian cohorts (HR = 1.28, p=0.12). This difference echoes the findings of Zhu et al. (64). Literature suggests that Asian prostate cancer cohorts differ in the prevalence of key driver mutations, such as TMPRSS2-ERG fusions and PTEN deletions, compared to Western populations (65). The lack of significance in Asian populations in our study might also stem from the limited number of studies (n=2) or differences in baseline inflammatory thresholds. Moreover, subgroup estimates for specific populations, including patients receiving combined therapy, American cohorts, and chemotherapy-treated patients assessed by PLR, were derived from very few studies with wide confidence intervals. Consequently, the observed associations ought to be viewed as preliminary and exploratory, serving primarily to inform future research rather than establishing definitive clinical thresholds in the absence of prospective verification. This observation necessitates caution when applying PLR cut-offs derived from Western literature to Asian patients and calls for region-specific validation studies. Furthermore, similar to the study by Guo et al., a major challenge for the clinical application of PLR is the lack of a unified cutoff value (66). Future research needs to determine the optimal PLR cutoff value for mCRPC in specific treatment contexts and populations based on large-scale prospective cohorts, using statistical methods such as receiver operating characteristic curves, to achieve more precise risk stratification. By addressing cutoff standardization and deepening the understanding of biological mechanisms, PLR has the potential to be combined with existing clinical and molecular markers to jointly construct a more comprehensive individualized prognostic and predictive model for mCRPC.

The LMR was the only marker where higher values predicted better survival (HR = 0.45, 95% CI: 0.31–0.67). This finding aligns with the role of monocytes and their tissue-resident macrophages in tumor progression. Monocytes are precursors to tumor-associated macrophages, which primarily polarize into the M2 phenotype with immunosuppressive properties in the tumor microenvironment, thereby promoting tumor growth, angiogenesis, and metastasis (67). Therefore, an elevated circulating monocyte count may indicate enhanced recruitment potential for tumor-associated macrophages, while a reduced lymphocyte count reflects impaired anti-tumor immune function. Compared to NLR and PLR, LMR reflects the inflammatory-immune balance from a complementary perspective, and its incorporation into clinical assessment may provide additional prognostic information.

SII, as a composite indicator integrating platelet, neutrophil, and lymphocyte counts, demonstrated the strongest prognostic association in mCRPC patients (pooled HR = 2.22, 95% CI: 1.86–2.66). This effect size exceeds that reported in mixed-stage prostate cancer meta-analyses, supporting the hypothesis that SII is particularly sensitive in the advanced mCRPC setting where inflammatory burden is maximal (68, 69). Recent real-world evidence suggests that SII may be especially valuable in identifying patients with “occult” high-risk disease who might benefit from intensified combination therapies rather than standard doublets (42). The superior discriminatory power of SII suggests it should be prioritized for inclusion in future multivariable prognostic nomograms.

Several potential sources contribute to the limitations and observed heterogeneity in this study. First, the lack of standardized cut-off values for markers like NLR and PLR often stems from variations in laboratory assay techniques, differences in the timing of blood sampling relative to treatment cycles, and the inherent biological variability of systemic inflammation across diverse patient populations. Second, the predominance of retrospective designs among the eligible articles inevitably makes the analysis susceptible to selection and information biases. These analyses frequently lack granular data on critical confounding factors that can significantly modulate peripheral blood counts. Lastly, the extensive study period spanning from 1998 to 2025 covers a transformative era in mCRPC management. Clinical heterogeneity across cohorts may stem from differences in prior treatments and therapy lines, driven by evolving standards of care.

The clinical significance of our subgroup analyses lies in validating these markers as versatile tools within the mCRPC therapeutic landscape. The remarkable consistency of NLR’s prognostic value across different ages, regions, and treatments, including chemotherapy and novel hormonal agents, establishes it as a robust “pan-treatment” risk stratification tool. This allows clinicians to identify high-risk phenotypes regardless of the therapeutic agent chosen. Conversely, the geographical disparity in PLR significance, which was observed in European but not Asian cohorts, provides a crucial clinical caution. This finding highlights the need for region-specific thresholds rather than universal cut-offs. Overall, integrating these accessible markers into routine assessment facilitates personalized prognostic counseling and informs clinical trial design. By identifying patients with high inflammatory burden, physicians can better select candidates for intensified therapies or closer monitoring, thereby advancing precision medicine in advanced prostate cancer.

Notwithstanding the strength of the pooled estimates, several inherent shortcomings warrant consideration. First, significant heterogeneity exists regarding cut-off values. While an NLR cut-off of 3.0 was common, variations across studies (ranging from 2.0 to 5.0) complicate the establishment of a universal clinical standard (70). Second, the majority of included studies were retrospective, introducing potential selection bias. While our sensitivity analysis confirmed the stability of the results, inherent biases in retrospective data collection cannot be fully eliminated. Third, data for LMR and SII were relatively scarce compared to NLR, limiting our ability to perform extensive subgroup analyses for these promising markers. Additionally, the software employed for meta-analysis (RevMan 5.4) does not support the generation of prediction intervals in forest plots, which limits a more comprehensive visual assessment of heterogeneity; future updates to this meta-analysis should consider incorporating prediction intervals using more flexible statistical platforms. Finally, we observed funnel plot asymmetry and significant Egger’s tests for NLR-related outcomes, suggesting potential small-study effects and selective publication. This may inflate the estimated adverse effect size of elevated NLR; therefore, while the direction of association is likely robust, the magnitude should be interpreted with caution and requires prospective validation.

To mitigate these constraints, subsequent investigations ought to focus on extensive, multi-institutional prospective cohorts designed to establish standardized and clinically validated cut-off values for immune-inflammatory indices. Such efforts are essential to ensure the reproducibility and comparability of results across different clinical settings. Additionally, future investigations should focus on the development of integrated prognostic nomograms that combine these blood-based markers with established clinicopathological features and emerging molecular biomarkers. Exploring the dynamic, longitudinal changes of these indices during treatment may also provide superior predictive value compared to single baseline measurements. Furthermore, more research is needed in non-Western populations to develop ethnicity-specific thresholds and validate the prognostic utility of less-studied markers such as SII and LMR. Finally, deep mechanistic studies are warranted to further elucidate how systemic inflammation drives treatment resistance and to identify potential therapeutic targets within the tumor-immune microenvironment.

## 5 Conclusion

This meta-analysis suggests that systemic inflammatory markers could hold significant prognostic value regarding the life expectancy of mCRPC patients. These accessible, low-cost biomarkers may, to some extent, capture key aspects of tumor-host immune interactions and provide meaningful prognostic information. While the associations observed across various clinical contexts suggest the potential utility of these markers for risk stratification, these findings should be interpreted with caution given the inherent limitations identified, such as heterogeneous cut-off values and the retrospective nature of most included studies. To advance the clinical integration of these promising biomarkers, subsequent investigations should prioritize prospective validation alongside the harmonization of measurement techniques and threshold values, while further investigating their predictive utility.

## Data Availability

The datasets presented in this study can be found in online repositories. The names of the repository/repositories and accession number(s) can be found in the article/**Supplementary Material**.

## References

[1] Sung H Ferlay J Siegel RL Laversanne M Soerjomataram I Jemal A . Global cancer statistics 2020: GLOBOCAN estimates of incidence and mortality worldwide for 36 cancers in 185 countries. CA Cancer J Clin. (2021) 71:209–49. doi:10.3322/caac.21660. PMID: 33538338

[2] Bray F Laversanne M Sung H Ferlay J Siegel RL Soerjomataram I . Global cancer statistics 2022: GLOBOCAN estimates of incidence and mortality worldwide for 36 cancers in 185 countries. CA Cancer J Clin. (2024) 74:229–63. doi:10.3322/caac.21834. PMID: 38572751

[3] Kulasegaran T Oliveira N . Metastatic castration-resistant prostate cancer: Advances in treatment and symptom management. Curr Treat Options Oncol. (2024) 25:914–31. doi:10.1007/s11864-024-01215-2. PMID: 38913213 PMC11236885

[4] Teo MY Rathkopf DE Kantoff P . Treatment of advanced prostate cancer. Annu Rev Med. (2019) 70:479–99. doi:10.1146/annurev-med-051517-011947. PMID: 30691365 PMC6441973

[5] Sartor O de Bono J Chi KN Fizazi K Herrmann K Rahbar K . Lutetium-177-PSMA-617 for metastatic castration-resistant prostate cancer. N Engl J Med. (2021) 385:1091–103. doi:10.1056/nejmoa2107322. PMID: 34161051 PMC8446332

[6] Cai M Song XL Li XA Chen M Guo J Yang DH . Current therapy and drug resistance in metastatic castration-resistant prostate cancer. Drug Resist Update. (2023) 68:100962. doi:10.1016/j.drup.2023.100962. PMID: 37068396

[7] de Visser KE Joyce JA . The evolving tumor microenvironment: From cancer initiation to metastatic outgrowth. Cancer Cell. (2023) 41:374–403. doi:10.1016/j.ccell.2023.02.016. PMID: 36917948

[8] Hibino S Kawazoe T Kasahara H Itoh S Ishimoto T Sakata-Yanagimoto M . Inflammation-induced tumorigenesis and metastasis. Int J Mol Sci. (2021) 22. doi:10.3390/ijms22115421. PMID: 34063828 PMC8196678

[9] Zapała P Garbas K Lewandowski Z Ślusarczyk A Ślusarczyk C Mielczarek Ł . Neutrophil-to-lymphocyte ratio predicts nodal involvement in unfavourable, clinically nonmetastatic prostate cancer patients and overall survival in pN1 patients. Sci Rep. (2023) 13:392. doi:10.1038/s41598-023-27542-2, PMID: 36624246 PMC9829873

[10] Templeton AJ McNamara MG Šeruga B Vera-Badillo FE Aneja P Ocaña A . Prognostic role of neutrophil-to-lymphocyte ratio in solid tumors: a systematic review and meta-analysis. J Natl Cancer Inst. (2014) 106:dju124. doi:10.1093/jnci/dju124. PMID: 24875653

[11] Guan Y Xiong H Feng Y Liao G Tong T Pang J . Revealing the prognostic landscape of neutrophil-to-lymphocyte ratio and platelet-to-lymphocyte ratio in metastatic castration-resistant prostate cancer patients treated with abiraterone or enzalutamide: a meta-analysis. Prostate Cancer Prostatic Dis. (2020) 23:220–31. doi:10.1038/s41391-020-0209-3. PMID: 32034294

[12] Wang H Yang R Liu D Li W . Association of pretreatment neutrophil-to-lymphocyte ratio with clinical outcomes in cancer immunotherapy: An evidence synthesis from 30 meta-analyses. Int Immunopharmacol. (2024) 132:111936. doi:10.1016/j.intimp.2024.111936. PMID: 38579566

[13] Ying J Zhou C Jin Y . Prognostic value of platelet to lymphocyte ratio in patients with castration-resistant prostate cancer: a systematic review and meta-analysis. Front Oncol. (2025) 15:1655520. doi:10.3389/fonc.2025.1655520. PMID: 41458591 PMC12739549

[14] Liao K Zhang X Liu J Teng F He Y Cheng J . The role of platelets in the regulation of tumor growth and metastasis: the mechanisms and targeted therapy. MedComm (2020). (2023) 4:e350. doi:10.1002/mco2.350. PMID: 37719444 PMC10501337

[15] Hu B Yang XR Xu Y Sun YF Sun C Guo W . Systemic immune-inflammation index predicts prognosis of patients after curative resection for hepatocellular carcinoma. Clin Cancer Res. (2014) 20:6212–22. doi:10.1158/1078-0432.ccr-14-0442. PMID: 25271081

[16] Kou J Huang J Li J Wu Z Ni L . Systemic immune-inflammation index predicts prognosis and responsiveness to immunotherapy in cancer patients: a systematic review and meta-analysis. Clin Exp Med. (2023) 23:3895–905. doi:10.1007/s10238-023-01035-y. PMID: 36966477

[17] Nishijima TF Muss HB Shachar SS Tamura K Takamatsu Y . Prognostic value of lymphocyte-to-monocyte ratio in patients with solid tumors: a systematic review and meta-analysis. Cancer Treat Rev. (2015) 41:971–8. doi:10.1016/j.ctrv.2015.10.003. PMID: 26481060

[18] Page MJ McKenzie JE Bossuyt PM Boutron I Hoffmann TC Mulrow CD . The PRISMA 2020 statement: an updated guideline for reporting systematic reviews. Bmj. (2021) 372:n71. doi:10.31222/osf.io/v7gm2. PMID: 33782057 PMC8005924

[19] Bauckneht M Rebuzzi SE Signori A Frantellizzi V Murianni V Lodi Rizzini E . The prognostic power of inflammatory indices and clinical factors in metastatic castration-resistant prostate cancer patients treated with radium-223 (BIO-Ra study). Eur J Nucl Med Mol Imaging. (2022) 49:1063–74. doi:10.1007/s00259-021-05550-6. PMID: 34486070 PMC8803683

[20] Gualdi-Russo E Zaccagni L . The Newcastle–Ottawa scale for assessing the quality of studies in systematic reviews. Publications. (2026) 14:4. doi:10.3390/publications14010004. PMID: 41725453

[21] Stang A . Critical evaluation of the Newcastle-Ottawa scale for the assessment of the quality of nonrandomized studies in meta-analyses. Eur J Epidemiol. (2010) 25:603–5. doi:10.1007/s10654-010-9491-z. PMID: 20652370

[22] Higgins JP Thompson SG Deeks JJ Altman DG . Measuring inconsistency in meta-analyses. Bmj. (2003) 327:557–62. doi:10.1136/bmj.327.7414.557. PMID: 12958120 PMC192859

[23] Boegemann M Schlack K Thomes S Steinestel J Rahbar K Semjonow A . The role of the neutrophil to lymphocyte ratio for survival outcomes in patients with metastatic castration-resistant prostate cancer treated with abiraterone. Int J Mol Sci. (2017) 18. doi:10.3390/ijms18020380. PMID: 28208664 PMC5343915

[24] Boegemann M Schlack K Früchtenicht L Steinestel J Schrader AJ Wennmann Y . A prognostic score for overall survival in patients treated with abiraterone in the pre- and post-chemotherapy setting. Oncotarget. (2019) 10:5082–91. doi:10.18632/oncotarget.27133. PMID: 31489117 PMC6707939

[25] Buttigliero C Pisano C Tucci M Vignani F Bertaglia V Iaconis D . Prognostic impact of pretreatment neutrophil-to-lymphocyte ratio in castration-resistant prostate cancer patients treated with first-line docetaxel. Acta Oncol. (2017) 56:555–62. doi:10.1080/0284186x.2016.1260772. PMID: 28068151

[26] Choi SY Ryu J You D Jeong IG Hong JH Ahn H . Prognostic factors of oncologic outcomes in metastatic chemotherapy-naïve castration-resistant prostate cancer treated with enzalutamide in actual clinical practice in East Asia. Urol Oncol. (2018) 36:401.e11–.e18. doi:10.1016/j.urolonc.2018.06.004. PMID: 30274641

[27] Chong W Zhang Z Luo R Gu J Lin J Wei Q . Integration of circulating tumor cell and neutrophil-lymphocyte ratio to identify high-risk metastatic castration-resistant prostate cancer patients. BMC Cancer. (2021) 21:655. doi:10.1186/s12885-021-08405-3. PMID: 34078304 PMC8170812

[28] Conteduca V Crabb SJ Jones RJ Caffo O Elliott T Scarpi E . Persistent neutrophil to lymphocyte ratio >3 during treatment with enzalutamide and clinical outcome in patients with castration-resistant prostate cancer. PloS One. (2016) 11:e0158952. doi:10.1371/journal.pone.0158952. PMID: 27434372 PMC4951050

[29] Conteduca V Caffo O Galli L Maugeri A Scarpi E Maines F . Association among metabolic syndrome, inflammation, and survival in prostate cancer. Urol Oncol. (2018) 36:240.e1–.e11. doi:10.1016/j.urolonc.2018.01.007. PMID: 29402534

[30] Conteduca V Scarpi E Matteucci F Caroli P Ravaglia G Fantini L . Multimodal approach to outcome prediction in metastatic castration-resistant prostate cancer by integrating functional imaging and plasma DNA analysis. JCO Precis Oncol. (2019) 3:1–13. doi:10.1200/po.18.00302. PMID: 35100689

[31] Donate-Moreno MJ Lorenzo-Sánchez MV Díaz de Mera-Sánchez Migallón I Herraiz-Raya L Esper-Rueda JA Legido-Gómez O . Inflammatory markers as prognostic factors in metastatic castration-resistant prostate cancer. Actas Urol Esp (Engl Ed). (2020) 44:692–700. doi:10.1016/j.acuroe.2020.11.009. PMID: 33010988

[32] España S Ochoa de Olza M Sala N Piulats JM Ferrandiz U Etxaniz O . PSA kinetics as prognostic markers of overall survival in patients with metastatic castration-resistant prostate cancer treated with abiraterone acetate. Cancer Manag Res. (2020) 12:10251–60. doi:10.2147/CMAR.S270392, PMID: 33116879 PMC7584507

[33] Fan L Wang R Chi C Cai W Zhang Y Qian H . Systemic immune-inflammation index predicts the combined clinical outcome after sequential therapy with abiraterone and docetaxel for metastatic castration-resistant prostate cancer patients. Prostate. (2018) 78:250–6. doi:10.1016/s1569-9056(18)31449-0. PMID: 29285775

[34] Fujiwara M Yuasa T Komai Y Numao N Yamamoto S Fukui I . Efficacy, prognostic factors, and safety profile of enzalutamide for non-metastatic and metastatic castration-resistant prostate cancer: a retrospective single-center analysis in Japan. Target Oncol. (2020) 15:635–43. doi:10.1007/s11523-020-00759-1. PMID: 33037973

[35] Jiang ZG Liao SG . Baseline neutrophil-lymphocyte ratio is associated with outcomes in patients with castration-resistant prostate cancer treated with docetaxel in South China. Med (Baltimore). (2021) 100:e27361. doi:10.1097/md.0000000000027361. PMID: 34596147 PMC8483836

[36] Koo KC Lee JS Ha JS Han KS Lee KS Hah YS . Optimal sequencing strategy using docetaxel and androgen receptor axis-targeted agents in patients with castration-resistant prostate cancer: utilization of neutrophil-to-lymphocyte ratio. World J Urol. (2019) 37:2375–84. doi:10.1016/s1569-9056(18)31451-9 30734074

[37] Linton A Pond G Clarke S Vardy J Galsky M Sonpavde G . Glasgow prognostic score as a prognostic factor in metastatic castration-resistant prostate cancer treated with docetaxel-based chemotherapy. Clin Genitourin Cancer. (2013) 11:423–30. doi:10.1016/j.clgc.2013.04.020. PMID: 23816526

[38] Lolli C Caffo O Scarpi E Aieta M Conteduca V Maines F . Systemic immune-inflammation index predicts the clinical outcome in patients with mCRPC treated with abiraterone. Front Pharmacol. (2016) 7:376. doi:10.3389/fphar.2016.00376. PMID: 27790145 PMC5062111

[39] Lorente D Mateo J Templeton AJ Zafeiriou Z Bianchini D Ferraldeschi R . Baseline neutrophil-lymphocyte ratio (NLR) is associated with survival and response to treatment with second-line chemotherapy for advanced prostate cancer independent of baseline steroid use. Ann Oncol. (2015) 26:750–5. doi:10.1093/annonc/mdu587. PMID: 25538172

[40] Man YN Chen YF . Systemic immune-inflammation index, serum albumin, and fibrinogen impact prognosis in castration-resistant prostate cancer patients treated with first-line docetaxel. Int Urol Nephrol. (2019) 51:2189–99. doi:10.1007/s11255-019-02265-4. PMID: 31456101

[41] Meisel A de Wit R Oudard S Sartor O Stenner-Liewen F Shun Z . Neutropenia, neutrophilia, and neutrophil-lymphocyte ratio as prognostic markers in patients with metastatic castration-resistant prostate cancer. Ther Adv Med Oncol. (2022) 14:17588359221100022. doi:10.1177/17588359221100022. PMID: 35677318 PMC9168856

[42] Neuberger M Goly N Skladny J Milczynski V Weiß C Wessels F . Systemic inflammatory biomarkers as predictive and prognostic factors in men with metastatic castration-refractory prostate cancer treated with docetaxel therapy: a comprehensive analysis in a German real-world cohort. J Cancer Res Clin Oncol. (2023) 149:3371–81. doi:10.1007/s00432-022-04220-w. PMID: 35939112 PMC10314827

[43] Nuhn P Vaghasia AM Goyal J Zhou XC Carducci MA Eisenberger MA . Association of pretreatment neutrophil-to-lymphocyte ratio (NLR) and overall survival (OS) in patients with metastatic castration-resistant prostate cancer (mCRPC) treated with first-line docetaxel. BJU Int. (2014) 114:E11–e17. doi:10.1111/bju.12531. PMID: 24529213 PMC4004702

[44] Onal C Sedef AM Kose F Oymak E Guler OC Sumbul AT . The hematologic parameters in metastatic castration-resistant prostate cancer patients treated with abiraterone acetate. Future Oncol. (2019) 15:1469–79. doi:10.2217/fon-2018-0635. PMID: 30977383

[45] Pei XQ He DL Tian G Lv W Jiang YM Wu DP . Prognostic factors of first-line docetaxel treatment in castration-resistant prostate cancer: roles of neutrophil-to-lymphocyte ratio in patients from Northwestern China. Int Urol Nephrol. (2017) 49:629–35. doi:10.1007/s11255-017-1524-z. PMID: 28161841

[46] Pisano C Tucci M RF DIS Turco F Samuelly A Bungaro M . Prognostic role of platelet-to-lymphocyte ratio and neutrophil-to-lymphocyte ratio in patients with metastatic castration resistant prostate cancer treated with abiraterone or enzalutamide. Minerva Urol Nephrol. (2021) 73:803–14. doi:10.23736/s2724-6051.21.04186-2. PMID: 33781017

[47] Şahin E Kefeli U Zorlu Ş Seyyar M Ozkorkmaz Akdag M Can Sanci P . Prognostic role of neutrophil-to-lymphocyte ratio, platelet-to-lymphocyte ratio, systemic immune-inflammation index, and pan-immune-inflammation value in metastatic castration-resistant prostate cancer patients who underwent 177Lu-PSMA-617. Med (Baltimore). (2023) 102:e35843. doi:10.1097/MD.0000000000035843, PMID: 38013293 PMC10681561

[48] Steffens F Wessels F Hetjens S Carl N Nitschke K Uysal D . Prognostic factors for overall survival in castration-resistant metastatic prostate cancer treated with docetaxel (MeProCSS): results from a German real-world cohort. Int Urol Nephrol. (2025) 57:2063–72. doi:10.1007/s11255-025-04389-2. PMID: 39871032 PMC12764561

[49] Sonpavde G Pond GR Armstrong AJ Clarke SJ Vardy JL Templeton AJ . Prognostic impact of the neutrophil-to-lymphocyte ratio in men with metastatic castration-resistant prostate cancer. Clin Genitourin Cancer. (2014) 12:317–24. doi:10.1016/j.clgc.2014.03.005. PMID: 24806399

[50] Templeton AJ Pezaro C Omlin A McNamara MG Leibowitz-Amit R Vera-Badillo FE . Simple prognostic score for metastatic castration-resistant prostate cancer with incorporation of neutrophil-to-lymphocyte ratio. Cancer. (2014) 120:3346–52. doi:10.1002/cncr.28890. PMID: 24995769

[51] Uemura K Kawahara T Yamashita D Jikuya R Abe K Tatenuma T . Neutrophil-to-lymphocyte ratio predicts prognosis in castration-resistant prostate cancer patients who received cabazitaxel chemotherapy. BioMed Res Int. (2017) 2017:7538647. doi:10.1155/2017/7538647. PMID: 28948170 PMC5602619

[52] Uzun M Gokcek S Kaya E Semiz HS . The prognostic role of systemic immune-inflammation index, SII, in metastatic castration-resistant prostate cancer patients. Discov Oncol. (2025) 16:317. doi:10.1007/s12672-025-02084-3. PMID: 40085163 PMC11908992

[53] van Soest RJ Templeton AJ Vera-Badillo FE Mercier F Sonpavde G Amir E . Neutrophil-to-lymphocyte ratio as a prognostic biomarker for men with metastatic castration-resistant prostate cancer receiving first-line chemotherapy: data from two randomized phase III trials. Ann Oncol. (2015) 26:743–9. doi:10.1093/annonc/mdu569. PMID: 25515657

[54] de Wit R Wülfing C Castellano D Kramer G Eymard JC Sternberg CN . Baseline neutrophil-to-lymphocyte ratio as a predictive and prognostic biomarker in patients with metastatic castration-resistant prostate cancer treated with cabazitaxel versus abiraterone or enzalutamide in the CARD study. ESMO Open. (2021) 6:100241. doi:10.1056/nejmoa1911206. PMID: 34450475 PMC8390550

[55] Wu KJ Pei XQ Tian G Wu DP Fan JH Jiang YM . PSA time to nadir as a prognostic factor of first-line docetaxel treatment in castration-resistant prostate cancer: evidence from patients in Northwestern China. Asian J Androl. (2018) 20:173–7. doi:10.4103/aja.aja_34_17. PMID: 28905815 PMC5858103

[56] Yamada Y Sakamoto S Rii J Yamamoto S Kamada S Imamura Y . Prognostic value of an inflammatory index for patients with metastatic castration-resistant prostate cancer. Prostate. (2020) 80:559–69. doi:10.1002/pros.23969. PMID: 32134137

[57] Yamamoto Y Ishii M Yoshimura A Hayashi T Kawamura N Nagahara A . Efficacy of cabazitaxel in patients with metastatic castration-resistant prostate cancer: a single-center study in Japan. Int J Urol. (2023) 30:20–7. doi:10.1111/iju.15052. PMID: 36168966

[58] Guo J Fang J Huang X Liu Y Yuan Y Zhang X . Prognostic role of neutrophil to lymphocyte ratio and platelet to lymphocyte ratio in prostate cancer: a meta-analysis of results from multivariate analysis. Int J Surg. (2018) 60:216–23. doi:10.1016/j.ijsu.2018.11.020. PMID: 30468905

[59] Zhang Y Zhou X Xu R Luo G Wang X . Neutrophil-to-lymphocyte ratio as a prognostic factor in patients with castration-resistant prostate cancer treated with docetaxel-based chemotherapy: a meta-analysis. BMC Urol. (2025) 25:17. doi:10.1186/s12894-024-01685-4. PMID: 39865253 PMC11770999

[60] Kawahara T Kato M Tabata K Kojima I Yamada H Kamihira O . A high neutrophil-to-lymphocyte ratio is a poor prognostic factor for castration-resistant prostate cancer patients who undergo abiraterone acetate or enzalutamide treatment. BMC Cancer. (2020) 20:919. doi:10.1186/s12885-020-07410-2. PMID: 32977754 PMC7519532

[61] Stangl-Kremser J Sun M Ho B Thomas J Nauseef JT Osborne JR . Prognostic value of neutrophil-to-lymphocyte ratio in patients with metastatic castration-resistant prostate cancer receiving prostate-specific membrane antigen targeted radionuclide therapy. Prostate. (2023) 83:1351–7. doi:10.1097/ju.0000000000003257.10. PMID: 37424145

[62] Albrengues J Shields MA Ng D Park CG Ambrico A Poindexter ME . Neutrophil extracellular traps produced during inflammation awaken dormant cancer cells in mice. Science. (2018) 361. doi:10.1126/science.aao4227. PMID: 30262472 PMC6777850

[63] Fridman WH Zitvogel L Sautès-Fridman C Kroemer G . The immune contexture in cancer prognosis and treatment. Nat Rev Clin Oncol. (2017) 14:717–34. doi:10.1038/nrclinonc.2017.101. PMID: 28741618

[64] Zhu Y Mo M Wei Y Wu J Pan J Freedland SJ . Epidemiology and genomics of prostate cancer in Asian men. Nat Rev Urol. (2021) 18:282–301. doi:10.1038/s41585-021-00442-8. PMID: 33692499

[65] Vlajnic T Bubendorf L . Molecular pathology of prostate cancer: a practical approach. Pathology. (2021) 53:36–43. doi:10.1016/j.pathol.2020.10.003. PMID: 33234230

[66] Guo W Lu X Liu Q Zhang T Li P Qiao W . Prognostic value of neutrophil-to-lymphocyte ratio and platelet-to-lymphocyte ratio for breast cancer patients: an updated meta-analysis of 17079 individuals. Cancer Med. (2019) 8:4135–48. doi:10.1002/cam4.2281. PMID: 31197958 PMC6675722

[67] Lin Y Xu J Lan H . Tumor-associated macrophages in tumor metastasis: biological roles and clinical therapeutic applications. J Hematol Oncol. (2019) 12:76. doi:10.1186/s13045-019-0760-3. PMID: 31300030 PMC6626377

[68] Qi W Zhou Y Liu Z Wang J Lv G Zhong M . Revealing the prognostic and clinicopathological significance of systemic immune-inflammation index in patients with different stage prostate cancer: a systematic review and meta-analysis. Front Med (Lausanne). (2022) 9:1052943. doi:10.3389/fmed.2022.1052943. PMID: 36388917 PMC9659961

[69] Meng L Yang Y Hu X Zhang R Li X . Prognostic value of the pretreatment systemic immune-inflammation index in patients with prostate cancer: a systematic review and meta-analysis. J Transl Med. (2023) 21:79. doi:10.1186/s12967-023-03924-y. PMID: 36739407 PMC9898902

[70] Islam MM Satici MO Eroglu SE . Unraveling the clinical significance and prognostic value of the neutrophil-to-lymphocyte ratio, platelet-to-lymphocyte ratio, systemic immune-inflammation index, systemic inflammation response index, and delta neutrophil index: an extensive literature review. Turk J Emerg Med. (2024) 24:8–19. doi:10.4103/tjem.tjem_198_23. PMID: 38343523 PMC10852137

