# Supplementary material for: Immune-inflammation index as prognostic markers in metastatic castration-resistant prostate cancer: a systematic review and meta-analysis
Source: Front Oncol. 2026 Apr 27;16:1806929. doi: 10.3389/fonc.2026.1806929 (PMC13158060; doi:10.3389/fonc.2026.1806929)
Supplement: Supplementary file 2 [file Table1.docx]

**Supplementary Table S1. Detailed search strategy in four databases**

**Pubmed-156**

((("Lymphocytes"[Mesh]) OR (((Lymphocyte) OR (Lymphoid Cells)) OR (Lymphoid Cell))) AND (Ratio OR index OR score)) AND (("Prostatic Neoplasms, Castration-Resistant"[Mesh]) OR (((((((((((((((((((((((((((((Castration-Resistant Prostatic Neoplasm) OR (Androgen-Independent Prostatic Neoplasms)) OR (Androgen Independent Prostatic Neoplasms)) OR (Androgen-Insensitive Prostatic Neoplasms)) OR (Androgen Insensitive Prostatic Neoplasms)) OR (Androgen-Resistant Prostatic Neoplasms)) OR (Androgen Resistant Prostatic Neoplasms)) OR (Castration-Resistant Prostatic Neoplasms)) OR (Castration Resistant Prostatic Neoplasms)) OR (Hormone Refractory Prostatic Neoplasms)) OR (Androgen-Independent Prostatic Neoplasm)) OR (Androgen-Insensitive Prostatic Neoplasm)) OR (Androgen-Resistant Prostatic Neoplasm)) OR (Castration-Resistant Prostatic Cancers)) OR (Androgen-Insensitive Prostatic Cancer)) OR (Androgen Insensitive Prostatic Cancer)) OR (Androgen-Resistant Prostatic Cancer)) OR (Androgen Resistant Prostatic Cancer)) OR (Castration-Resistant Prostatic Cancer)) OR (Castration Resistant Prostatic Cancer)) OR (Hormone Refractory Prostatic Cancer)) OR (Androgen-Independent Prostatic Cancer)) OR (Androgen Independent Prostatic Cancer)) OR (Androgen-Insensitive Prostatic Cancers)) OR (Androgen-Resistant Prostatic Cancers)) OR (Androgen-Independent Prostatic Cancers)) OR (CRPC)) OR (Castration Resistant Prostate Cancer)) OR (Castration-Resistant Prostate Cancer)))

**Embase-465**

((Lymphocytes or (Lymphocyte or Lymphoid Cells or Lymphoid Cell)) and (Ratio or index or score) and (Prostatic Neoplasms, Castration-Resistant or (Castration-Resistant Prostatic Neoplasm or Androgen-Independent Prostatic Neoplasms or Androgen Independent Prostatic Neoplasms or Androgen-Insensitive Prostatic Neoplasms or Androgen Insensitive Prostatic Neoplasms or Androgen-Resistant Prostatic Neoplasms or Androgen Resistant Prostatic Neoplasms or Castration-Resistant Prostatic Neoplasms or Castration Resistant Prostatic Neoplasms or Hormone Refractory Prostatic Neoplasms or Androgen-Independent Prostatic Neoplasm or Androgen-Insensitive Prostatic Neoplasm or Androgen-Resistant Prostatic Neoplasm or Castration-Resistant Prostatic Cancers or Androgen-Insensitive Prostatic Cancer or Androgen Insensitive Prostatic Cancer or Androgen-Resistant Prostatic Cancer or Androgen Resistant Prostatic Cancer or Castration-Resistant Prostatic Cancer or Castration Resistant Prostatic Cancer or Hormone Refractory Prostatic Cancer or Androgen-Independent Prostatic Cancer or Androgen Independent Prostatic Cancer or Androgen-Insensitive Prostatic Cancers or Androgen-Resistant Prostatic Cancers or Androgen-Independent Prostatic Cancers or CRPC or Castration Resistant Prostate Cancer or Castration-Resistant Prostate Cancer))).af.

**Cochrane-53**

((Lymphocytes or (Lymphocyte or Lymphoid Cells or Lymphoid Cell)) and (Ratio or index or score) and (Prostatic Neoplasms, Castration-Resistant or (Castration-Resistant Prostatic Neoplasm or Androgen-Independent Prostatic Neoplasms or Androgen Independent Prostatic Neoplasms or Androgen-Insensitive Prostatic Neoplasms or Androgen Insensitive Prostatic Neoplasms or Androgen-Resistant Prostatic Neoplasms or Androgen Resistant Prostatic Neoplasms or Castration-Resistant Prostatic Neoplasms or Castration Resistant Prostatic Neoplasms or Hormone Refractory Prostatic Neoplasms or Androgen-Independent Prostatic Neoplasm or Androgen-Insensitive Prostatic Neoplasm or Androgen-Resistant Prostatic Neoplasm or Castration-Resistant Prostatic Cancers or Androgen-Insensitive Prostatic Cancer or Androgen Insensitive Prostatic Cancer or Androgen-Resistant Prostatic Cancer or Androgen Resistant Prostatic Cancer or Castration-Resistant Prostatic Cancer or Castration Resistant Prostatic Cancer or Hormone Refractory Prostatic Cancer or Androgen-Independent Prostatic Cancer or Androgen Independent Prostatic Cancer or Androgen-Insensitive Prostatic Cancers or Androgen-Resistant Prostatic Cancers or Androgen-Independent Prostatic Cancers or CRPC or Castration Resistant Prostate Cancer or Castration-Resistant Prostate Cancer))).af.

**WOS-169**

(((Lymphocytes) OR (((Lymphocyte) OR (Lymphoid Cells)) OR (Lymphoid Cell))) AND (Ratio OR index OR score)) AND ((Prostatic Neoplasms, Castration-Resistant) OR (((((((((((((((((((((((((((((Castration-Resistant Prostatic Neoplasm) OR (Androgen-Independent Prostatic Neoplasms)) OR (Androgen Independent Prostatic Neoplasms)) OR (Androgen-Insensitive Prostatic Neoplasms)) OR (Androgen Insensitive Prostatic Neoplasms)) OR (Androgen-Resistant Prostatic Neoplasms)) OR (Androgen Resistant Prostatic Neoplasms)) OR (Castration-Resistant Prostatic Neoplasms)) OR (Castration Resistant Prostatic Neoplasms)) OR (Hormone Refractory Prostatic Neoplasms)) OR (Androgen-Independent Prostatic Neoplasm)) OR (Androgen-Insensitive Prostatic Neoplasm)) OR (Androgen-Resistant Prostatic Neoplasm)) OR (Castration-Resistant Prostatic Cancers)) OR (Androgen-Insensitive Prostatic Cancer)) OR (Androgen Insensitive Prostatic Cancer)) OR (Androgen-Resistant Prostatic Cancer)) OR (Androgen Resistant Prostatic Cancer)) OR (Castration-Resistant Prostatic Cancer)) OR (Castration Resistant Prostatic Cancer)) OR (Hormone Refractory Prostatic Cancer)) OR (Androgen-Independent Prostatic Cancer)) OR (Androgen Independent Prostatic Cancer)) OR (Androgen-Insensitive Prostatic Cancers)) OR (Androgen-Resistant Prostatic Cancers)) OR (Androgen-Independent Prostatic Cancers)) OR (CRPC)) OR (Castration Resistant Prostate Cancer)) OR (Castration-Resistant Prostate Cancer))) (Topic)
