# Supplementary material for: Immune-inflammation index as prognostic markers in metastatic castration-resistant prostate cancer: a systematic review and meta-analysis
Source: Front Oncol. 2026 Apr 27;16:1806929. doi: 10.3389/fonc.2026.1806929 (PMC13158060; doi:10.3389/fonc.2026.1806929)
Supplement: Supplementary file 3 [file Table2.docx]

| **Table S2. Quality evaluation of the eligible studies with Newcastle–Ottawa scale.** | | | | | | | | | |  |
| --- | --- | --- | --- | --- | --- | --- | --- | --- | --- | --- |
| Study | Selection | | | | Comparability | | Outcome | | |  |
|  | Representative-ness | Selection of  non-exposed | Ascertainment  of exposure | Outcome not present at start | Comparability on most important factors | Comparability on other risk factors | Assessment of outcome | Long enough follow-up (median≥1 year) | Adequacy  (completeness) of follow-up |  |
| Linton 2013 | * | * | * | * | * | * | * | - | * |  |
| Nuhn 2014 | * | * | * | * | * | - | * | * | * |  |
| Sonpavde 2014 | * | * | * | * | * | * | * | * | * |  |
| Templeton 2014 | * | - | * | * | * | * | * | * | * |  |
| van Soest 2014 | * | - | * | * | * | - | * | * | * |  |
| Lorente 2015 | * | - | * | * | * | - | * | * | * |  |
| Lolli 2016 | * | - | * | * | * | - | * | * | * |  |
| Conteduca 2016 | - | - | * | * | * | - | * | * | * |  |
| Uemura 2017 | * | - | * | * | * | - | * | - | * |  |
| Buttigliero 2017 | * | * | * | * | * | * | * | * | * |  |
| Boegemann 2017 | * | - | * | * | * | - | * | * | * |  |
| Fan 2017 | - | - | * | * | * | - | * | * | * |  |
| Pei 2017 | * | * | * | * | * | - | * | * | * |  |
| Wu 2018 | * | - | * | * | * | - | * | * | * |  |
| Onal 2018 | * | - | * | * | * | - | * | * | * |  |
| Conteduca 2018 | * | * | * | * | * | - | * | * | * |  |
| Choi 2018 | * | - | * | * | * | - | * | * | * |  |
| Boegemann 2019 | * | - | * | * | * | - | * | * | * |  |
| Conteduca 2019 | - | - | * | * | * | - | * | * | * |  |
| Koo 2019 | * | * | * | * | * | - | * | * | * |  |
| Man 2019 | * | * | * | * | * | - | * | * | * |  |
| Donate-Moreno 2020 | * | * | * | * | * | - | * | * | * |  |
| España 2020 | * | - | * | * | - | - | * | * | * |  |
| Yamada 2020 | * | - | * | * | * | - | * | * | * |  |
| Fujiwara 2020 | * | * | * | * | * | - | * | * | * |  |
| Bauckneht 2021 | * | - | * | * | * | - | * | * | * |  |
| Chong 2021 | * | * | * | * | - | - | * | - | * |  |
| Wit 2021 | * | * | * | * | * | - | * | * | * |  |
| Jiang 2021 | - | * | * | * | * | - | * | * | * |  |
| Pisano 2021 | - | - | * | * | * | - | * | * | * |  |
| Meisel 2022 | | * | * | * | * | * | - | * | * | * |
| Neuberger 2022 | * | - | * | * | - | - | * | * | * |  |
| Sahin 2023 | * | * | * | * | * | - | * | * | * |  |
| Yamamoto 2023 | * | - | * | * | * | - | * | - | * |  |
| Steffens 2025 | * | * | * | * | * | - | * | * | * |  |
| Uzun 2025 | * | * | * | * | - | - | * | * | * |  |
| *Indicates criterion met; - indicates significant of criterion not met. | | | | | | | | | |  |
